# Dental Floss Holders

**Published:** 1874-09

**Authors:** 


### DENTAL FLOSS HOLDERS.

This is a little instrument for holding and using silk floss for cleansing between the teeth. It renders this hitherto difficult, and unpleasant process, an easy and agreeable one, and in no respect is it more objectionable than the tooth-pick, and it accomplishes a work that the ordinary pick can not do. The want for some appliance of this kind has for a long time been recognized, and the want is fully supplied by the introduction of the Floss Holder.

It contains from six to ten yards of floss, and is of such form as to be carried in the pocket with as little inconvenience as an ordinary tooth pick.

Every dentist should have them on hand, to supply those patients who desire to take the best care of their teeth. They are manufactured by Codman and Shurtleff of Boston, and we presume can be obtained at any of the dental depots.

